# Associations between adiposity indicators and composite motor fitness among boys and girls in late childhood

**DOI:** 10.3389/fspor.2026.1979414

**Published:** 2026-10-06

**Authors:** Leona Roca Badrić, Marko Badrić, Igor Vrga, Dario Novak

**Affiliations:** 1 Faculty of Teacher Education, University of Zagreb, Zagreb, Croatia; 2 General Hospital Sisak, Sisak, Croatia; 3 Faculty of Kinesiology, University of Zagreb, Zagreb, Croatia

**Keywords:** adiposity, body composition, body fat percentage, children, motor fitness, waist-to-height ratio

## Abstract

**Background:**

Adiposity is associated with physical and motor fitness in children, but the relative contribution of different body-composition and adiposity indicators to overall motor fitness remains unclear.

**Objective:**

This study examined the associations of body mass index (BMI), body fat percentage (BF%), fat mass (FM), muscle mass (MM), and waist-to-height ratio (WHtR) with a composite motor fitness index (MOTOFIT) in younger school-age children.

**Methods:**

A cross-sectional study was conducted in 306 children (163 girls and 143 boys; mean age 9.89 ± 0.60 years). Body composition was assessed using bioelectrical impedance analysis. Motor fitness was evaluated using hand tapping, standing long jump, sit-ups, and the 10 × 5 m shuttle run. Standardized Z-scores from the four tests were combined into MOTOFIT. Associations were examined using Pearson correlations and separate hierarchical multiple regression models adjusted for age and sex, while differences across adiposity-status groups were analyzed using ANCOVA.

**Results:**

BF% showed the strongest negative association with MOTOFIT (*r* = −0.252, *p* < 0.001), followed by WHtR (*r* = −0.209, *p* < 0.001), FM (*r* = −0.205, *p* < 0.001), and BMI (*r* = −0.170, *p* = 0.003), whereas MM was not significantly associated with MOTOFIT (*r* = −0.009, *p* = 0.875). After adjustment for age and sex, BF% provided the largest incremental contribution to the explained variance in MOTOFIT (*Δ*R^2^ = 0.057), followed by WHtR (*Δ*R^2^ = 0.051), FM (*Δ*R^2^ = 0.042), and BMI (*Δ*R^2^ = 0.037), whereas MM did not significantly contribute to the model. Adiposity status was significantly associated with MOTOFIT (*F*(2,301) = 11.764, *p* < 0.001, *η*p^2^ = 0.072). Children in the normal body fat group had higher adjusted MOTOFIT values than children with elevated body fat (*p* = 0.004) and those with obesity (*p* < 0.001).

**Conclusion:**

Higher adiposity was associated with lower overall motor fitness in late childhood. BF% and WHtR provided greater incremental information on MOTOFIT than BMI, whereas MM did not independently contribute to the model. These findings support the complementary assessment of total and central adiposity alongside multidimensional motor fitness when evaluating physical development in children.

## 1 Introduction

Physical fitness is an important marker of health and functional capacity during childhood and adolescence. Higher levels of physical fitness are associated with more favorable body composition, greater physical activity, better cardiometabolic health, and a lower risk of adverse health outcomes later in life (1–3). Recent evidence from an overview of systematic reviews and meta-analyses including more than 125,000 observations further demonstrates that physical fitness, particularly cardiorespiratory and muscular fitness, is consistently associated with a broad range of health outcomes in children and adolescents (4). Because childhood represents a critical period for the development of both body composition and physical fitness, identifying factors associated with less favorable fitness profiles may contribute to the early recognition of children at increased health risk.

Among the factors associated with physical and motor fitness in children, adiposity has received considerable attention. Excess adipose tissue may impair motor performance by increasing the non-functional mass that must be displaced during locomotor and weight-bearing activities, increasing the energetic cost of movement, and reducing strength and power relative to total body mass (5, 6). These effects may be particularly relevant for tasks involving jumping, acceleration, rapid changes of direction, and repeated displacement of the body. Systematic reviews consistently indicate that children with overweight or obesity tend to perform worse in several components of physical and motor fitness, including explosive strength, speed, agility, and other weight-bearing motor tasks (5, 7). Recent findings in younger children similarly indicate differences in physical fitness and motor skills according to weight status and associations between body-composition characteristics and fitness performance (8). In Croatian children of a similar age, higher adiposity has also been associated with lower cardiorespiratory fitness, with body fat percentage showing a significant negative association with cardiorespiratory fitness in both sexes (9).

However, the relationship between adiposity and motor fitness may depend substantially on how adiposity is quantified. Body mass index (BMI) remains the most widely used anthropometric indicator in pediatric epidemiology because it is inexpensive, simple to calculate, and suitable for population-level surveillance. Nevertheless, BMI does not distinguish fat mass (FM) from fat-free mass (FFM) and provides limited information regarding the distribution of adipose tissue (10, 11). Consequently, children with similar BMI values may have substantially different body-composition profiles. Indicators that more directly characterize body composition, such as body fat percentage (BF%), FM, and muscle mass (MM), or central adiposity, such as waist-to-height ratio (WHtR), may therefore provide complementary information beyond BMI when examining the relationship between body composition and physical functioning.

Distinguishing adipose from metabolically and functionally active tissue may be particularly important for understanding motor performance. Adipose tissue contributes to the mass that must be displaced during weight-bearing movement but does not contribute proportionally to force generation, whereas skeletal muscle contributes directly to force production and motor performance. Evidence from pediatric populations suggests that fat and non-fat body compartments may therefore show different relationships with physical fitness. Henriksson et al. found that a higher fat mass index was associated with poorer cardiorespiratory fitness, lower-body muscular strength, and motor fitness, whereas a higher fat-free mass index was associated with better performance across several fitness domains (12). Although these findings were obtained in preschool children and were based on FFM rather than muscle mass specifically, they support the broader concept that adipose and non-adipose body compartments may have contrasting functional relationships with physical fitness.

Evidence from school-aged children further supports the value of assessing body composition in addition to conventional anthropometry. Hsu et al., in children with a mean age of approximately 10 years, simultaneously examined BMI z-score, WHtR, BF%, and muscle weight in relation to several components of physical fitness (13). BF% was independently associated with endurance running and sit-up performance, while muscle weight was positively associated with sit-up performance. These findings suggest that adipose and muscular components of body composition may provide complementary information regarding children's fitness profiles and that their functional implications may differ according to the fitness component examined.

An additional methodological consideration is that motor fitness is a multidimensional construct encompassing several capacities, including muscular strength and endurance, speed, agility, coordination, and other aspects of motor performance (14, 15). Standardized field-based fitness batteries have been developed to capture complementary dimensions of health-related fitness in young people (16), while evidence also supports the health relevance of muscular fitness as a distinct component of youth fitness (17). Assessing individual motor tests separately may therefore provide only a partial representation of a child's overall motor fitness. Standardizing and combining performance across several motor domains into a composite index may provide a broader representation of motor functioning and facilitate examination of the overall relationship between body composition and motor fitness (14, 18). Such an approach is particularly relevant when comparing adiposity indicators because individual fitness tests may differ in the extent to which they are influenced by body mass and body composition.

Despite growing evidence linking adiposity and body composition with physical fitness in children, important questions remain. Previous studies have commonly examined BMI or individual body-composition indicators in relation to separate fitness outcomes, whereas comparatively little is known about the relative contribution of multiple body-composition and adiposity indicators to overall multidimensional motor fitness (10, 11, 13, 14). Moreover, it remains unclear whether BF%, FM, MM, and WHtR provide additional information regarding motor fitness compared with BMI when age and sex are taken into account. This distinction has practical relevance because BMI is widely used in pediatric, school, and public-health settings, whereas complementary measures of total and central adiposity and muscle mass may provide a more comprehensive characterization of children's body composition in relation to functional performance.

Therefore, the primary aim of this study was to examine the associations of BMI, BF%, FM, MM, and WHtR with a composite motor fitness index (MOTOFIT) in younger school-age children. Secondary aims were to determine whether MOTOFIT differed according to adiposity status and to assess the independent contribution of each body-composition and adiposity indicator to the explained variance in MOTOFIT after controlling for age and sex. Based on previous evidence, we hypothesized that higher adiposity would be associated with lower composite motor fitness and that BF% and WHtR would show stronger negative associations with MOTOFIT than BMI. Given the functional role of skeletal muscle in motor performance, we hypothesized that muscle mass would not show the negative association with composite motor fitness expected for the adiposity indicators.

## 2 Materials and methods

### 2.1 Study design and participants

This cross-sectional observational study included 306 younger school-age students (163 girls and 143 boys) aged 9 to 11 years (mean age, 9.89 ± 0.60 years) from 11 primary schools located in the City of Zagreb, Zagreb County, and Sisak-Moslavina County in the continental part of the Republic of Croatia, representing predominantly urban and semi-urban settings. Participating schools were selected from the study regions based on their accessibility for study implementation. School principals were contacted in advance by written correspondence or e-mail to obtain institutional approval. All schools initially contacted agreed to participate; consequently, no additional schools were approached. The exact number of parental consent forms distributed was not systematically recorded, and therefore the parental response rate could not be calculated. Eligible participants regularly attended Physical Education classes, had no diagnosed health conditions or injuries that could affect motor-test performance, and had written informed consent provided by a parent or legal guardian. Students who did not complete all planned measurements or did not meet the inclusion criteria were excluded from the analyses.

The study was conducted during the 2024/2025 school year. All procedures were performed during regular Physical Education classes according to a predefined and standardized protocol that was applied consistently across participating schools. The study was planned, conducted, and reported in accordance with the Strengthening the Reporting of Observational Studies in Epidemiology (STROBE) guidelines (19).

Data from this cohort have previously been used to investigate the association between physical fitness and health-related quality of life (20). The present study addresses a distinct research question by examining the associations of body composition and adiposity indicators with composite motor fitness. The primary outcome (MOTOFIT), adiposity-focused analyses, adiposity-status classification, and statistical models reported in the present study were not examined in the previous publication.

The research was conducted in accordance with the ethical principles of the Declaration of Helsinki and was approved by the Ethics Committee of the Faculty of Teacher Education, University of Zagreb (Class: 641-03/24-01/02; Reg. No.: 251-378-01-24-2). Before data collection, parents or legal guardians of all participants provided written informed consent for their children's participation.

### 2.2 Data collection procedures

Data collection was conducted during two regular Physical Education class periods in elementary-school sports halls. Anthropometric measurements and body composition assessment were performed on the first testing day, whereas motor fitness testing was performed on the second day. All measurements were conducted in the morning by the same research team according to a pre-standardized protocol to ensure consistency of procedures and comparability of results. Before motor testing, all participants completed a standardized warm-up of approximately 10 min consisting of light jogging, dynamic stretching, and preparatory motor tasks.

### 2.3 Anthropometric and body composition assessment

Anthropometric measurements were performed in the morning according to standardized anthropometric procedures (21). Body height was measured using a portable SECA 213 stadiometer (Seca GmbH & Co. KG, Hamburg, Germany). Body mass and body composition were assessed using a Tanita DC-360 P bioelectrical impedance analyzer (Tanita Corporation, Tokyo, Japan). Fasting status was not standardized, and food and fluid intake prior to BIA measurement was not systematically controlled.

Body mass (kg), body fat percentage (BF%), fat mass (FM, kg), and muscle mass (MM, kg) were recorded. Body mass index (BMI) was calculated as body mass in kilograms divided by height in meters squared (kg/m^2^). Waist circumference was measured using a non-stretchable anthropometric tape at the natural waistline (the narrowest part of the trunk between the lower ribs and the superior border of the iliac crest), with the participant standing upright, arms relaxed, abdomen relaxed, and breathing normally. All waist circumference measurements were performed by the same trained researcher using the standardized protocol described above. Measurements were not repeated for the assessment of intra-rater reliability; therefore, an intra-rater ICC was not available. Because only one researcher performed these measurements, inter-rater reliability was not applicable. Waist-to-height ratio (WHtR) was calculated as waist circumference (cm) divided by body height (cm).

### 2.4 Adiposity classification

Adiposity status was determined using age- and sex-specific reference values for BF% according to McCarthy et al. (22). For the present analyses, participants were classified into three groups: normal body fat (≤85th percentile), elevated body fat (>85th to <95th percentile), and obesity (≥95th percentile). Children with BF% values below the 2nd percentile, classified as underfat in the original reference curves, were included in the normal body fat group for the present analyses. Accordingly, the term ‘obesity’ used in the present study refers specifically to the BF%-based category defined by the McCarthy et al. reference curves and should not be interpreted as a clinical diagnosis of obesity or as a BMI-based weight-status classification.

### 2.5 Motor fitness assessment

Motor fitness was assessed using four field-based motor tests: hand tapping, standing long jump, sit-ups, and the 10 × 5 m shuttle run. The standing long jump, sit-ups, and 10 × 5 m shuttle run were administered according to standardized EUROFIT procedures (23), whereas hand tapping was performed according to the protocol described by Findak et al. (24). The four tests were selected because they are feasible for standardized administration during regular Physical Education classes and collectively assess complementary motor capacities relevant to school-age children: speed of alternating movements (hand tapping), lower-body explosive power (standing long jump), repetitive trunk muscular strength/endurance (sit-ups), and agility/change-of-direction speed (10 × 5 m shuttle run). The selection was informed by established school- and field-based fitness assessment frameworks. Standing long jump and shuttle-run tasks, in particular, are widely represented in European youth fitness batteries, including ALPHA, HELENA, and PREFIT (16, 25, 26), whereas hand tapping and sit-ups were derived from established standardized motor assessment protocols (23, 24). The present battery was intended to provide a multidimensional field-based representation of motor fitness rather than a comprehensive assessment of all motor fitness domains. Accordingly, flexibility, balance, and predominantly upper-body strength were not directly assessed.

Hand tapping assessed the speed of alternating movements of the dominant hand. Performance was expressed as the number of correctly completed double taps within 15 s, with a higher score indicating better performance. The standing long jump assessed explosive power of the lower extremities, and the best result from two attempts was retained for analysis. Sit-ups assessed repetitive strength of the trunk musculature, with performance expressed as the number of correctly completed repetitions. The 10 × 5 m shuttle run assessed agility, with performance expressed as the total completion time in seconds; a lower time indicated better performance (23).

EUROFIT-based field testing remains widely used in pediatric fitness research, and standardized field-based batteries facilitate reliable assessment and comparison of fitness components across populations (1, 14, 16). All motor tests were conducted in primary-school sports halls under standardized conditions.

### 2.6 Composite motor fitness index (MOTOFIT)

To integrate performance across the four motor tests into a single indicator of overall motor fitness, a composite motor fitness index (MOTOFIT) was calculated. Because the tests were expressed in different measurement units, individual results were standardized using Z-scores, thereby allowing them to be combined into a common metric (27). MOTOFIT was treated as a study-derived composite score rather than as an externally validated scale.

For each motor test, a standardized Z-score was calculated as z = (x−x̄)/SD. Because lower completion time indicated better performance in the 10 × 5 m shuttle run, its standardized Z-score was multiplied by −1. Thus, higher standardized scores consistently represented better performance across all four tests. MOTOFIT was calculated as the arithmetic mean of the four standardized Z-scores, with each motor-test component receiving equal weight. Higher MOTOFIT values indicated better overall motor fitness.

### 2.7 Statistical analysis

Continuous variables are presented as mean and standard deviation (M ± SD), whereas categorical variables are presented as absolute and relative frequencies. Where appropriate, effect-size estimates and 95% confidence intervals (CI) were reported in addition to statistical significance. Sex differences were examined using independent-samples Student's t-tests, and Cohen's d was calculated as an effect-size estimate. Homogeneity of variances was assessed using Levene's test; when the assumption of equal variances was not met, Welch's correction was applied. Associations between body composition and adiposity indicators and MOTOFIT were examined using Pearson's correlation coefficients.

The independent contribution of individual body composition and adiposity indicators to MOTOFIT was examined using separate hierarchical multiple regression models. Separate models were used because the body composition and adiposity indicators represent related and partly overlapping aspects of body size and composition; their simultaneous inclusion in a single model could therefore introduce multicollinearity and complicate interpretation of their individual associations with MOTOFIT. In Step 1 of each model, age and sex were entered as control variables. In Step 2, one body composition or adiposity indicator (BMI, BF%, FM, MM, or WHtR) was entered individually. Multicollinearity within each regression model was assessed using tolerance and variance inflation factor (VIF) values. Unstandardized regression coefficients (B), standard errors (SE), standardized regression coefficients (*β*), t-values, 95% CIs, coefficients of determination (R^2^), and changes in explained variance (*Δ*R^2^) were reported. Assumptions relevant to the applied parametric analyses, including normality, linearity, homoscedasticity, independence of residuals, and multicollinearity, were examined before inferential analyses. Distributional characteristics were assessed using skewness and kurtosis. Given the sample size (*N* = 306), absolute skewness values <2 and kurtosis values < 7 were considered indicative of no substantial departure from normality (28). Across the analyzed continuous variables, skewness ranged from −0.175 to 1.658 and kurtosis from −0.319 to 3.936, indicating no substantial departures from normality. Differences in MOTOFIT among adiposity-status groups were examined using analysis of covariance (ANCOVA), with age and sex included as covariates. Homogeneity of variances was assessed using Levene's test, and Bonferroni-adjusted pairwise comparisons were used to examine between-group differences. Effect size was estimated using partial eta squared (*η*p^2^). Statistical analyses were performed using IBM SPSS Statistics for Windows, version 29.0 (IBM Corp., Armonk, NY, USA). Statistical significance was set at *p* < 0.05, and exact *p*-values were reported where appropriate.

## 3 Results

Table 1 shows descriptive indicators of anthropometric characteristics, adiposity indicators and motor fitness for the entire sample of 306 students. The mean age of the participants was 9.89 ± 0.60 years. The average values of BMI, body fat percentage and WHtR were 18.83 ± 4.20 kg/m^2^, 20.73 ± 8.85% and 0.46 ± 0.06, respectively. The study-derived composite motor fitness index (MOTOFIT) had a mean of 0.00 ± 0.63 and ranged from −1.66 to 1.56.

**Table 1:** Descriptive indicators of anthropometric and motor variables for the entire sample (*N* = 306).

Variable	M	SD	Min.	Max
Age (years)	9.89	0.60	9	11
Body height (cm)	145.31	8.23	124.00	168.00
Body weight (kg)	40.56	11.96	22.50	85.60
BMI (kg/m^2^)	18.83	4.20	10.80	34.20
Body fat percentage (BF%)	20.73	8.85	3.00	43.10
Waist circumference (cm)	66.14	10.44	35.00	98.00
WHtR	0.46	0.06	0.25	0.65
Fat mass (kg)	9.48	6.95	0.80	36.60
Muscle mass (MM, kg)	30.50	9.24	13.40	69.00
Taping (number)	22.02	3.86	14.00	32.00
Long jump from standing position (cm)	138.25	20.63	85.00	195.00
Sit-ups (number)	32.33	8.19	13.00	54.00
Running 10 × 5 m (s)	26.31	3.85	10.40	38.17
Composite index of motor fitness (MOTOFIT)	0.00	0.63	−1.66	1.56

M, arithmetic mean; SD, standard deviation; Min*,* minimum value; Max*,* maximum value; BMI*,* body mass index; WHtR*,* waist-to-height ratio; MOTOFIT*,* composite motor fitness index.

The results in Table 2 show sex differences in adiposity and motor fitness indicators. Boys had significantly higher BMI (*p* = 0.021) and MM (*p* = 0.003), and performed better in the standing long jump (*p* = 0.037) and MOTOFIT (*p* = 0.008) than girls. No statistically significant sex differences were observed for BF%, FM, WHtR, hand tapping, sit-ups, or the 10 × 5 m shuttle run (all *p* > 0.05). Effect sizes for the statistically significant differences were small (Cohen's d = 0.24–0.35).

**Table 2:** Differences between boys and girls in indicators of body composition and adiposity and motor fitness.

Variable	Girls (*n* = 163) M ± SD	Boys (*n* = 143) M ± SD	t	*p*	Cohen's d
BMI	18.31 ± 3.89	19.42 ± 4.47*	−2.31	.021	−0.27
Body fat (%)	21.58 ± 8.83	19.76 ± 8.81	1.80	.073	0.21
Fat mass (kg)	9.50 ± 6.69	9.45 ± 7.25	0.06	.952	0.01
Muscle mass (MM, kg)	29.03 ± 8.90	32.18 ± 9.37**	−3.00	.003	−0.35
WHtR	0.45 ± 0.06	0.46 ± 0.06	−1.91	.057	−0.22
Taping (number)	21.63 ± 3.90	22.48 ± 3.77	−1.94	.054	−0.22
Long jump from a standing position	135.94 ± 19.94	140.88 ± 21.15*	−2.09	.037	−0.24
Sit-ups (number)	32.00 ± 8.25	32.71 ± 8.13	−0.76	.448	−0.09
Running 10 × 5 m (s)	26.67 ± 3.96	25.90 ± 3.70	1.75	.082	0.20
MOTOFIT	−0.09 ± 0.62	0.10 ± 0.62**	−2.68	.008	−0.31

M, arithmetic mean; SD, standard deviation; BMI*,* body mass index; WHtR*,* waist-to-height ratio; MOTOFIT, composite motor fitness index. * *p* < 0.05; ** *p* < 0.01; *** *p* < 0.001. Welch–Satterthwaite degrees of freedom correction was applied when the assumption of homogeneity of variance was not met.

Table 3 shows the results of Pearson's correlation analyses between body composition and adiposity indicators, individual motor tests, and the composite motor fitness index (MOTOFIT). Higher adiposity was generally associated with poorer performance in weight-bearing motor tests and with lower MOTOFIT values. The strongest negative correlation with MOTOFIT was observed for body fat percentage (*r* = −0.252, *p* < 0.001), followed by WHtR (*r* = −0.209, *p* < 0.001), fat mass (*r* = −0.205, *p* < 0.001), and BMI (r = −0.170, *p* = 0.003). MM was not significantly associated with MOTOFIT (*r* = −0.009, *p* = 0.875). Among the individual motor tests, the standing long jump showed the most consistent negative associations with adiposity indicators.

**Table 3:** Pearson's correlation coefficient between body composition and adiposity indicators and motor fitness.

Variable	BMI	BF (%)	FM (kg)	MM (kg)	WHtR
Hand taping	−.005	−.063	−.022	.140*	−.012
Long jump from a standing position	−.258**	−.298**	−.309**	−.107	−.168**
Sit−ups	−.145*	−.172**	−.145*	.148**	−.157**
Running 10 × 5 m	.039	.117*	.060	.222**	.196**
MOTOFIT	−.170**	−.252**	−.205**	−.009	−.209**

BMI, body mass index; BF, body fat percentage; FM, fat mass; MM, muscle mass; WHtR, waist-to-height ratio; MOTOFIT, composite motor fitness index; r, Pearson's correlation coefficient. * *p* < 0.05; ** *p* < 0.01; *** *p* < 0.001.

Table 4 presents the results of the hierarchical multiple regression analyses. The base model, which included age and sex, explained 2.3% of the variance in MOTOFIT [R^2^ = 0.023, adjusted R^2^ = 0.017; F(2,303) = 3.626, *p* = 0.028]. When entered separately in Step 2, BMI, BF%, FM, and WHtR each significantly increased the explained variance in MOTOFIT, whereas muscle mass did not. BF% provided the largest incremental contribution to the explained variance (*Δ*R^2^ = 0.057), followed by WHtR (*Δ*R^2^ = 0.051), FM (*Δ*R^2^ = 0.042), and BMI (*Δ*R^2^ = 0.037).

**Table 4:** Hierarchical regression analysis of body composition and adiposity indicators as predictors of the composite motor fitness index (MOTOFIT).

Adiposity indicator	B	SE	*β*	t	p	95% CI for B	*Δ*R^2^	R^2^
BMI	−.029	.008	−.193	−3.424	<.001***	−.045 to −.012	.037	.060
BF%	−.017	.004	−.240	−4.316	<.001***	−.025 to −.009	.057	.080
FM	−.018	.005	−.204	−3.663	<.001***	−.028 to −.009	.042	.065
MM	−.002	.004	−.034	−0.564	.573	−.010 to.006	.001	.024
WHtR	−2.295	.561	−.228	−4.089	<.001***	−3.399 to −1.191	.051	.075

B, unstandardized regression coefficient; SE, standard error; β, standardized regression coefficient; t, t-value; CI, confidence interval; *Δ*R^2^, change in the coefficient of determination after including each indicator of body composition and adiposity; R^2^, coefficient of determination of the model; BMI, body mass index; BF%, body fat percentage; FM, fat mass; MM, muscle mass; WHtR, waist-to-height ratio. The basic model included age and sex [R^2^ = 0.023; adjusted R^2^ = 0.017; F(2,303) = 3.626; *p* = 0.028]. * *p* < 0.05; ** *p* < 0.01; *** *p* < 0.001; Multicollinearity diagnostics indicated no problematic collinearity within the individual regression models (tolerance = 0.899–0.997; VIF = 1.003–1.113).

Table 5 presents the results of the analysis of covariance (ANCOVA). Levene's test indicated that the assumption of homogeneity of variance was met [F(2,303) = 0.226; *p* = 0.798]. ANCOVA showed a statistically significant effect of adiposity status on MOTOFIT after adjustment for age and sex [F(2,301) = 11.764; *p* < 0.001; *η*p^2^ = 0.072]. Bonferroni-adjusted pairwise comparisons showed that children in the normal body fat group had significantly higher adjusted MOTOFIT values than children with elevated body fat (*p* = 0.004) and children with obesity (*p* < 0.001). No statistically significant difference was observed between the elevated body fat and obesity groups (*p* = 1.000). Age was not a significant covariate [F(1,301) = 0.040; *p* = 0.842], whereas sex had a statistically significant effect on MOTOFIT [F(1,301) = 8.769; *p* = 0.003; *η*p^2^ = 0.028].

**Table 5:** Differences in the composite motor fitness index according to adiposity status after adjustment for age and sex (ANCOVA).

Adiposity status	N	Estimated mean ± SE	95% CI	Bonferroni *post hoc* comparisons
Normal body fat	226	0.099 ± 0.040	0.021 to 0.178	Normal > Elevated BF (*p* = 0.004**)
Elevated body fat	29	−0.289 ± 0.112	−0.509 to −0.069	Normal > Obesity (*p* < 0.001***)
Obesity	51	−0.275 ± 0.084	−0.441 to −0.110	Elevated BF = Obesity (*p* = 1.000)

Estimated means represent estimated marginal means adjusted for age and sex. SE, standard error; CI, confidence interval; MOTOFIT, composite motor fitness index. Pairwise comparisons were adjusted using the Bonferroni correction. ** *p* < 0.01; *** *p* < 0.001.

## 4 Discussion

The present study examined the associations of BMI, body fat percentage (BF%), fat mass (FM), muscle mass (MM), and waist-to-height ratio (WHtR) with the composite motor fitness index (MOTOFIT) in younger school-age children. The main findings were that higher adiposity was associated with lower overall motor fitness and that the strength of this relationship differed across adiposity indicators. BF% showed the strongest negative association with MOTOFIT and provided the largest incremental contribution to the explained variance after adjustment for age and sex, followed by WHtR, FM, and BMI. In contrast, MM was not significantly associated with MOTOFIT and did not provide a significant additional contribution to the regression model. In addition, children in the normal body fat group had higher adjusted MOTOFIT values than children with elevated body fat and those with obesity. Together, these findings suggest that indicators reflecting the amount and distribution of adipose tissue may provide additional information on children's motor fitness beyond that obtained from BMI alone.

The inverse association between adiposity and motor performance is consistent with previous research showing that children with higher adiposity or overweight/obesity generally perform less favorably in several components of physical and motor fitness (5, 29–31). In the present study, the standing long jump showed the most consistent negative associations with adiposity indicators among the individual motor tests, while weaker but significant associations were also observed for sit-ups and the 10 × 5 m shuttle run. This pattern suggests that adiposity may be particularly disadvantageous for tasks in which performance depends on generating force or power relative to body mass and efficiently displacing the body. Similar findings have been reported in systematic reviews and pediatric studies emphasizing poorer performance in explosive strength, speed, agility, and other weight-bearing tasks among children with greater adiposity (5, 7, 29, 30).

Gajewska et al. (32) observed a similar pattern in a somewhat older sample of adolescents aged 13-14 years, where a higher proportion of body fat was associated with poorer performance in selected EUROFIT fitness components. Although the latter sample was older than the children in the present study, these findings support the broader interpretation that greater adiposity may compromise motor performance, particularly in tasks requiring rapid or forceful movement of body mass. Excess adipose tissue can increase the mechanical and energetic cost of movement and reduce relative strength and power, which provides a plausible explanation for the observed pattern (5, 7).

One of the central contributions of the present study is the direct comparison of several body-composition and adiposity indicators within the same analytical framework. After adjustment for age and sex, BF% provided the largest incremental contribution to MOTOFIT, followed by WHtR, FM, and BMI. However, the absolute incremental contributions of these adiposity indicators were modest, accounting for an additional 3.7% to 5.7% of the variance in MOTOFIT beyond age and sex. Thus, although adiposity was significantly associated with composite motor fitness, it explained only a relatively small proportion of its variability, indicating that motor fitness is likely influenced by multiple additional biological, behavioral, and contextual factors. This pattern is consistent with growing evidence that BMI, although practical and useful for surveillance, is limited as a stand-alone indicator because it does not distinguish adipose from non-adipose tissue and does not capture fat distribution (33–36). In younger populations, reliance on BMI alone may therefore lead to misclassification or incomplete characterization of body composition, particularly among children with relatively greater muscularity or atypical body-composition patterns (34–36). The present findings do not diminish the value of BMI for population monitoring; rather, they indicate that more direct or complementary indicators may improve the characterization of adiposity in relation to functional motor performance.

The relatively strong contribution of WHtR is also noteworthy. WHtR reflects central adiposity and has been proposed as a simple anthropometric indicator of cardiometabolic risk in pediatric populations (37–40). More recent evidence indicates that WHtR may better approximate fat-mass-related risk than BMI in some pediatric contexts. Agbaje (33), for example, reported better longitudinal agreement of WHtR with DXA-measured fat mass than BMI, while Kim et al. (41) also demonstrated the usefulness of waist-height ratio in relation to obesity and cardiometabolic risk. In the present study, WHtR provided a greater incremental contribution to MOTOFIT than BMI, suggesting that central adiposity may have functional relevance in addition to its established cardiometabolic significance.

FM was also negatively associated with MOTOFIT and significantly increased the explained variance beyond age and sex, although its incremental contribution was smaller than that of BF% and WHtR. This distinction may reflect the fact that absolute FM is influenced by overall body size, whereas BF% expresses adiposity relative to total body mass. In growing children, relative and distributional indicators may therefore characterize the functional burden of adiposity differently from absolute FM. The present pattern supports the use of multiple, conceptually distinct indicators when examining body composition in relation to motor fitness rather than treating BMI, FM, BF%, and WHtR as interchangeable measures.

Unlike the adiposity indicators, MM did not make a statistically significant additional contribution to explaining the variance in MOTOFIT after adjustment for age and sex. This finding suggests that absolute muscle mass alone may not necessarily translate into better overall motor fitness, particularly when performance involves moving one's own body mass. Motor performance depends not only on the quantity of muscle tissue but also on muscle quality, neuromuscular activation, coordination, technique, and force and power relative to total body mass. Hsu et al. (13) reported a positive association between muscle weight and sit-up performance in school-aged children, illustrating that muscle mass may be more closely related to specific muscular tasks than to a multidimensional composite outcome. Saraiva et al. (30) similarly showed that higher adiposity was associated with poorer motor skills and physical fitness regardless of lean soft tissue, further suggesting that the functional burden of adiposity may remain important even when non-fat tissue is taken into account. Thus, the present result should not be interpreted as evidence that skeletal muscle is unimportant for performance; rather, absolute MM may be insufficient to capture the functional characteristics of muscle relevant to performance across heterogeneous motor tasks.

Sex differences provide additional context for these findings. Boys had higher MM and BMI and performed better in the standing long jump and MOTOFIT than girls, whereas no statistically significant sex differences were observed for BF%, FM, WHtR, hand tapping, sit-ups, or the 10 × 5 m shuttle run. These findings are broadly consistent with reports showing better performance among boys in selected components of explosive strength, power, agility, and overall fitness during childhood (42, 43). One possible contributor is a higher level of habitual moderate-to-vigorous physical activity among boys, as reported across multiple countries (44). Biological maturation may also contribute to between-child differences in body composition and motor performance, even within the relatively narrow chronological age range examined in the present study. Children of the same chronological age may differ substantially in maturational timing and status, which can influence body composition, muscle mass, strength, power, and other aspects of physical performance (45). Because biological maturation and habitual physical activity were not directly assessed in the present study, their potential contribution to the observed sex differences and adiposity–motor fitness associations could not be determined. Therefore, these findings should be interpreted cautiously, and future studies should consider incorporating indicators of biological maturation when examining relationships between adiposity and motor fitness in late childhood.

The use of the standardized composite MOTOFIT index enabled the integration of results from different motor tests into a single indicator of overall motor fitness. Because motor fitness is multidimensional, a composite approach allows several aspects of motor functioning to be considered simultaneously without giving greater weight to a test merely because of its measurement scale (14, 18). In the present study, this approach facilitated comparison of the relative contribution of different body-composition and adiposity indicators to overall motor fitness. The finding that BF% and WHtR provided greater incremental contributions than BMI is consistent with previous evidence emphasizing the potential value of body-composition and fat-distribution measures when explaining fitness outcomes in school-aged children (13, 46), and further supports the limitations of BMI as a stand-alone marker (33, 41). Recent research in Frontiers in Sports and Active Living also demonstrates that different sport backgrounds may be associated with distinct profiles of basic motor skills and physical fitness in pre- and early-pubertal children, reinforcing the value of a multidimensional perspective on motor functioning (47). Nevertheless, MOTOFIT is a study-derived composite index rather than an externally validated scale, and its interpretation should remain specific to the present analytical framework until its measurement properties are examined in independent samples. The observed associations should not be interpreted as indicating that higher adiposity uniformly impairs all dimensions of motor fitness. The strength and direction of the associations varied across individual motor tasks, with the most consistent negative associations observed for weight-bearing performance, particularly the standing long jump. Moreover, given the cross-sectional design, the direction of these relationships cannot be established. Greater adiposity may increase the mechanical and energetic demands of weight-bearing movement and thereby constrain performance, whereas lower motor fitness and reduced engagement in physical activity may, over time, contribute to greater adiposity. These pathways are not mutually exclusive and may operate bidirectionally.

The ANCOVA findings complement the correlational and regression analyses. After adjustment for age and sex, children in the normal body fat group had significantly higher MOTOFIT values than children with elevated body fat and those with obesity, whereas no significant difference was observed between the elevated body fat and obesity groups. This pattern is consistent with previous studies showing generally poorer motor and physical fitness among children with higher adiposity (5, 29, 31). Importantly, the group-based analysis did not indicate a clear progressive decline in motor fitness across the three BF%-based adiposity categories. Although children in both the elevated body fat and obesity groups had significantly lower adjusted MOTOFIT values than those in the normal body fat group, the adjusted means were similar in the elevated body fat and obesity groups, and the difference between these two groups was not statistically significant. Thus, the findings are more consistent with a distinction between children with normal BF% and those with higher BF% than with a graded dose–response pattern across the three adiposity categories. However, given the relatively small number of participants in the elevated body fat group, this pattern should not be interpreted as evidence of a specific adiposity threshold for reduced motor fitness.

From a practical perspective, the findings support the use of complementary body-composition and motor-fitness information in school and field settings. BMI remains a simple and useful population-level measure, but waist circumference and WHtR can be obtained inexpensively and non-invasively and may provide additional information on central adiposity, while BF% may offer further information on total adiposity when appropriate equipment and standardized procedures are available. These measures should be regarded as complementary assessment tools rather than diagnostic criteria. Similarly, multidimensional motor-fitness assessment may provide physical education teachers, researchers, and health professionals with a broader profile of children's functional status than any single motor test. At the group level, repeated assessment of complementary adiposity indicators together with motor-fitness measures may help characterize developmental patterns, identify groups of children who may benefit from additional opportunities to develop motor fitness, and inform the planning and evaluation of inclusive physical activity programs. This integrated perspective is supported by school-based intervention research showing that physical activity programs can influence movement behaviors, aerobic fitness, motor competence, and body-composition-related outcomes over time (48). The present findings therefore support strategies that simultaneously promote favorable body composition and the development of diverse motor-fitness capacities rather than focusing exclusively on body weight or a single fitness component. For future research, combining multiple adiposity indicators with multidimensional motor-fitness assessment may also provide a more comprehensive framework for examining how changes in body composition relate to changes in specific and overall motor performance over time.

Several strengths of the present study should be acknowledged. The sample included more than 300 younger school-age children, multiple complementary indicators of body composition and adiposity were examined within the same analytical framework, and motor fitness was assessed using standardized field tests representing different motor domains. Separate hierarchical regression models allowed the incremental contribution of each indicator to MOTOFIT to be evaluated after adjustment for age and sex, while the adiposity-status analysis provided a complementary group-based perspective.

The study also has limitations. First, the cross-sectional design precludes causal inference, and bidirectional relationships between adiposity and motor fitness are plausible. Second, body composition was assessed using bioelectrical impedance analysis rather than a reference method such as dual-energy x-ray absorptiometry. BIA-derived estimates can be influenced by hydration and other measurement conditions. Fasting status was not standardized, and food and fluid intake prior to BIA measurement was not systematically controlled. Therefore, BF%, FM, and MM should be interpreted as device-derived estimates. Third, potentially relevant factors such as habitual physical activity, biological maturation, dietary behavior, and socioeconomic and environmental characteristics were not included in the analyses. In particular, opportunities for school- and out-of-school physical activity, participation in organized sport, and characteristics of the school and community environment may influence both adiposity and motor fitness. Although the participating schools represented predominantly urban and semi-urban settings, these contextual factors were not systematically assessed and their potential contribution to the observed associations could therefore not be examined. Future studies should incorporate individual-, family-, school-, and community-level contextual factors and consider potential differences across urban and rural environments. Fourth, participating schools were selected based on their accessibility for study implementation and were located in the continental part of Croatia, predominantly in urban and semi-urban settings. Therefore, the sample should not be considered nationally representative, and the findings may not be generalizable to children from other geographic, socioeconomic, or rural settings. Fifth, MOTOFIT was derived from four field tests and has not yet been externally validated as a composite measure. Although MOTOFIT integrated four complementary motor capacities, the test battery did not include direct assessments of flexibility, balance, or predominantly upper-body strength. Several of the included tasks also involved displacement of body mass and emphasized whole-body or lower-body performance. Consequently, the observed associations with adiposity may partly reflect the greater mechanical demands imposed by higher adiposity during weight-bearing motor tasks and should not be generalized to all dimensions of motor fitness. Future studies should include a broader range of motor tasks to determine whether associations with adiposity differ across specific motor fitness domains. Future longitudinal and intervention studies should examine whether changes in BF%, central adiposity, and muscle-related characteristics predict changes in multidimensional motor fitness and should evaluate the reproducibility and predictive validity of composite motor-fitness indices.

## 5 Conclusion

The findings of this study indicate that higher adiposity is associated with lower overall motor fitness in younger school-age children. Among the examined adiposity indicators, body fat percentage (BF%) provided the largest incremental contribution to the explained variance in the composite motor fitness index (MOTOFIT), followed by waist-to-height ratio (WHtR), fat mass (FM), and BMI, whereas muscle mass (MM) did not significantly contribute to the model. Children in the normal body fat group had higher overall motor fitness than children with elevated body fat and those with obesity after adjustment for age and sex. These findings suggest that indicators that more directly reflect the amount and distribution of adipose tissue may provide additional information on the relationship between adiposity and motor fitness beyond that provided by BMI alone. The results support the complementary assessment of body composition and multidimensional motor fitness when examining and monitoring physical development in late childhood.

## Data Availability

The original contributions presented in the study are included in the article/Supplementary Material, further inquiries can be directed to the corresponding author/s.
